# An Immunosensing System Using Stilbene Glycoside as a Fluorogenic Substrate for an Enzymatic Reaction Model

**DOI:** 10.3390/s8095661

**Published:** 2008-09-15

**Authors:** Xue-Hui Zhan, Fu-Chun Gong, Shu-Zhen Tan, Peng-Mian Huang, Ya-Fei Tan

**Affiliations:** College of Chemistry and Biological Engineering, Changsha University of Science and Technology, Changsha, 410076, Hunan, P.R. China

**Keywords:** Stilbene glycoside, HRP fluorogenic substrate, enzyme-linked immunosensing, *Brucella melitensis*

## Abstract

A natural product, stilbene glycoside (2,3,5,4′-tetrahydroxy-diphenylethylene-2-*O*-glucoside, TBG), has been evaluated for the first time as a potential substrate for horseradish peroxidase (HRP)-catalyzed fluorogenic reactions. The properties of TBG as a fluorogenic substrate for HRP and its application in a fluorometric enzyme-linked immunosensing system were compared with commercially available substrates such as *p*-hydroxyphenylpropionic acid (pHPPA), chavicol and Amplex red using *Brucella melitensis* antibody (BrAb) as a model analyte. The immunosensing body based on HRP-BrAb was constructed by dispersing graphite, BrAg and paraffin wax at room temperature. In a competitive immunoassay procedure, the BrAb competed with HRP-BrAb to react with the immobilized BrAg. In the enzymatic reaction, the binding HRP-BrAb on the sensing body surface can catalyze the polymerization reaction of TBG by H_2_O_2_ forming fluorescent dimers and causing an increase in fluorescence intensity. TBG showed comparable ability for HRP detection and its enzyme-linked immunosensing reaction system, in a linear detection ranging of 3.5×10^−8^∼7.6×10^−6^g/L and with a detection limit of 1.7×10^−9^ g/L. The immobilized biocomposite surface could be regenerated with excellent reproducibility (RSD=3.8%) by simply polishing with an alumina paper. The proposed immunosensing system has been used to determine the BrAb in rabbit serum samples with satisfactory results.

## 1. Introduction

Enzyme-linked immunosorbent assays (ELISA), introduced in the 20th century in early seventies and coupling the amplification effect of enzymatic reactions with selective antigen-antibody binding can provide sensitivity comparable with that of radioimmunoassay (RIA) [1, 2]. Several enzymes, such as alkaline phosphatase (AP), b-d-galactosidse and horseradish peroxidase (HRP) may be used as labels. HRP is one of the two most widely used enzyme labels in medical diagnostics and research applications, the other being alkaline phosphatase (AP). HRP is often applied in immunoassays and nucleic acid hybridization assays, in part because of the ready availability of peroxidase-conjugated antibodies to haptens such as biotin, fluorescein isothiocyanate, and digoxigenin [3]. The detection of HRP-label content can be carried out in various ways, including photometric, fluorimetric, chemiluminometric or electrochemical techniques after enzymatic conversion of a suitable substrate into a detectable product [4]. The key point of the ELISA method is the use of an appropriate substrate for the HRP detection. Thus, if a more feasible substrate for enzymatic reaction and a more sensitive method for enzymatic product quantitation is employed, it should be possible to detect lower level of enzyme labels and to shorten the duration of the enzyme catalysis step of the assay.

Generally, the selectivity of immunoassays is given by the immune components (parts of or entire antibody), and the sensitivity is often limited by the detection technique used. The excellent sensitivity and wide linear range typical of fluorometric detection have attracted attention in recent years, with the development of FELISA. It is essential that an appropriate fluorogenic substrate is employed for HRP detection in the FELISA methods.

The adaptation to HRP was attempted by Zapata and co-workers using 4-hydroxybenzoic acid (HBA) [5]. The oxidation products of 4-hydroxyphenylpropionic acid (pHPPA) [6], chavicol [7], Amplex red [8] and prochlorperazine [9] did not sensitize their fluorescence measurements in the corresponding experiment setup. The other common drawbacks of aforementioned fluorogenic substrates are their insufficient reaction rates, relatively low stability in air or toward H_2_O_2_ in the absence of HRP, tending to cause strong background signals and poor water-solubility [10, 11]. Work in our laboratory has demonstrated that the use of a suitable fluorogenic substrate for HRP makes possible the detection of enzyme reaction products at much lower levels than can be detected using the aforementioned substrates with conventional determination techniques.

**Figure 1.**
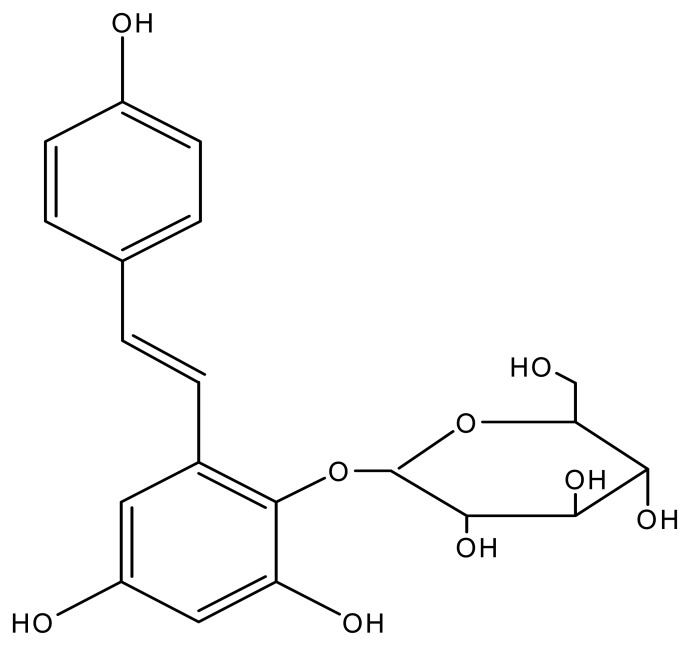
Chemical structure of stilbene glycoside.

In this work, a natural lipid-lowering drug, stilbene glycoside (2,3,5,4′-tetrahydroxy diphenylethylene-2-*O*-glucoside, TBG, Figure 1) was evaluated for the first time as a fluorogenic substrate for HRP-catalyzed reactions and applied an enzyme-linked immunosensing system using *Brucella melitensis* antibody (BrAb) as a model analyte. The properties of TBG as a potential fluorogenic substrate for HRP in a FELISA method were compared with commercially available substrates such as *p*-hydroxyphenylpropionic acid (pHPPA), chavicol and Amplex red in the BrAg-based immunosensing system. An immunocomposite support body was prepared by dispersion of *Brucella melitensis* antigens (BrAg), graphite and paraffin wax at a low temperature. The surface of the immobilized BrAg biocomposites can be renewed by polishing the used biocomposites layer, and the resulting surface serves as a platform for the competitive immunoreaction and HRP-enzymatic reaction. In the enzymatic reaction, HRP catalyzes the conversion of TBG into strongly fluorescent dimers. The content of HRP-BrAb attached to the aforementioned immunocomposite surface is proportional to the fluorescence intensity of enzymatic product. In the utilization of the novel substrate TBG, the proposed approach allows to detect lower level of enzyme labels for BrAb assay. The proposed BrAg-graphite-paraffin based immunosensing system has been applied to determination of BrAb in rabbit serum samples using a competitive binding assay with the aid of HRP-BrAb.

## 2. Experimental Section

### 2.1. Materials and Reagents

Horseradish peroxidase (HRP), stilbene glycoside (2,3,5,4′-tetrahydroxydiphenylethylene -2-*O*-glucoside, TBG), *p*-hydroxyphenylpropionic acid (pHPPA), chavicol and Amplex red were obtained from Sigma. H_2_O_2_ and bovine serum albumin (BSA) were purchased from Shanghai Reagents (Shanghai, P.R. China). *Brucella melitensis* antibody (BrAb) and *Brucella melitensis* antigen (BrAg) were gifts from Hunan Agricultural University. All other reagents were of analytical reagent grade, and triply distilled water was used throughout.

### Preparation of stilbene glycoside stock solution (1×10^−3^ mol/L)

Stilbene glycoside (3.56 mg) was dissolved in a small volume of water. To this solution, a B-R buffer (a 0.01M Na_2_HPO_4_−0.01 M NaH_2_PO_4_ solution, pH 5.8) is added to a final volume of 100 mL. This solution is diluted with the same B-R buffer when in use. The other substrate solutions were prepared by the same procedure.

### 2.2. Apparatus

Fluorescence measurements were performed on a RF-5000 fluorescence spectrophotometer (Japan). UV-visible spectroscopy experiments were carried out on a Shimadzu UV-1601 PC spectrophotometer (Japan). A model CSS501 thermostat (Chongqing, P.R. China) was employed to control the incubation temperature. All fluorescence measurements were performed at room temperature.

### 2.3. Stability measurement of stilbene glycoside

To TBG stock solutions (1 mL), pH 5.8 B-R solutions were added to a final volume of 10 mL and these were then subjected to the treatment and fluorescence experiments as follows: (1) The resulting solutions are stored at room temperature for 4, 8, 12, 24, 48 h, 5 and 10 d, respectively; (2) the aforementioned solutions are exposed to a temperature of 25, 30, 35, 45, 50, and 60 °C for 30 min, respectively; (3) to the aforementioned B-R buffer solutions, 3.00×10^−4^mol/L H_2_O_2_-1×10^−6^ mol/L HRP-BrAb and different concentrations of TBG were added. After a 2 min-reaction, the resulting solutions were subjected to fluorescence measurement.

### 2.4. Preparation of HRP-BrAb conjugate

The conjugation was performed according to a modification of a reported method [12]. An appropriate HRP (15 mg dissolved in 1.0 mL of 0.1 mol acetate buffer at pH 6.8) was combined with 0.1 mol NaIO_4_ (0.20 mL) and incubated for 25 min at 4 °C. Glycol (0.5 mL of 2.5% solution, v/v) was added to the solution and incubated for an additional 30 min at room temperature. BrAb (5 mg) was added and the pH of the solution was adjusted to 9.0 with a sodium carbonate buffer solution (pH 9.5). The resulting solution was allowed to stand overnight at 4 °C. NaHB_4_ (0.1 mL of 5 mg/mL solution) was slowly added and allowed to incubate 2 h at 4 °C. The resulting solution was dialyzed against a 0.1 mol phosphate buffer (pH 7.4) overnight at 4 °C. Further purification was conducted by gel filtration on Sepharose G-25 column to give the HRP-BrAb conjugates. The enzymatic activity unit of HRP-BrAb determined by UV-spectroscopy was 1140U/mg.

### 2.5. Immobilization of BrAg

**Figure 2.**
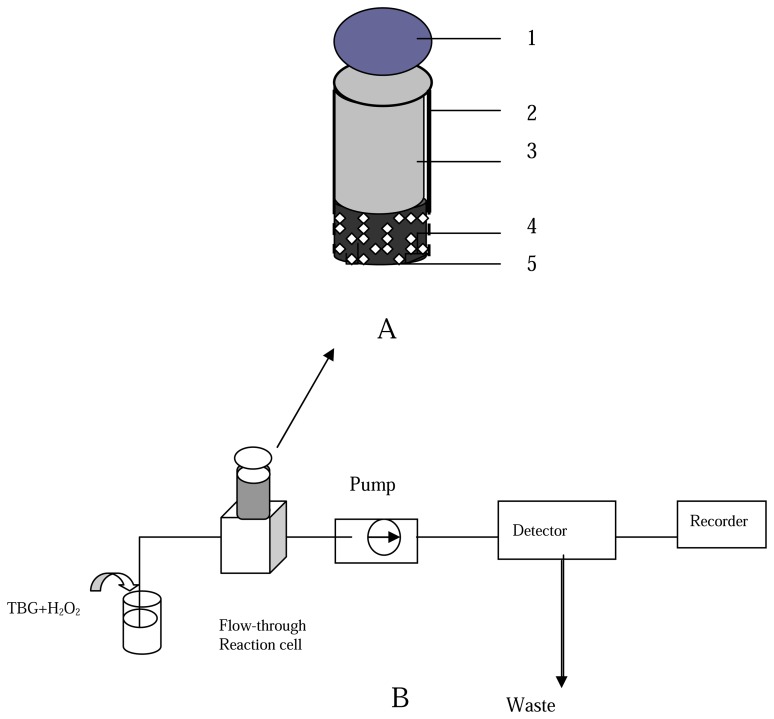
A: Configuration of the BrAg-based immunosensing device. (1) PVC nut, (2) PVC tube, (3) PVC screw, (4) BrAg-paraffix-graphite matrix, (5) entrapped BrAg. B: Schematic diagram of flow injection system coupling with fluometry.

The BrAg based immunosening body was prepared according to the method reported elsewhere [13] with slight modifications. A BrAg-BSA-modified graphite matrix was prepared as follows: BrAg (5 mg) and an appropriate amount of BSA (16 mg) were dissolved in a cold B-R buffer solution (4 °C, 1 mL), and the solution was mixed with graphite powder (2.5 g). The mixture was left to dry in a desiccator at 4 °C. The BrAg-BSA-modified graphite and the paraffin wax dissolved in THF were thoroughly mixed for a paraffin wax to carbon weight ratio of 2:3. The resulting paste squeezed into the PVC tube of 6 mm i.d to a depth of 1 cm. When not in use, the BrAg-supported body were stored in a dry state at 4 °C. The configuration of the BrAg-modified immunosensing body is shown in Figure 2 A.

### 2.6. Renewal of the surface of the supported immunoreagent body

The surface of supported immunoreagent could be renewed by turning the nut to extrude 0.1 mm-thick outer paste layer and polishing with an alumina paper (0.5 μm) to produce a smooth, shiny surface. The supported immunocomposite surface was finally cleaned with doubly distilled water.

### 2.7. BrAb Measurement procedure

The analytical procedure for BrAb is as follows: in the first step, the regenerated immunocomposite surface was immersed into a B-R solution (pH 5.8, 3 mL), which consists of 0.1% B-R (w/v), different amount of BrAb as the analyte solution and HRP-BrAb conjugate. After a 30-min incubation when the free BrAb competed with the HRP-BrAb conjugate for binding sites on the antigenes at 37 °C, the incubated immunocomposite support body was rinsed three times with a washing solution (0.1 mol/L Tris-HCl-KCl, pH 7.5) to remove the excess of free BrAb and HRP-BrAb conjugate attached to the immunocomposite surface. Next, the incubated immunocomposite support body with bound BrAb-HRP was mounted into the flow-through reaction cell in the path of the flowing stream. The schematic diagram of flow injection system coupling with fluorimetry for BrAb assay is shown in Figure 2B.

In the enzymatic reaction procedure, a substrate solution containing TBG (1.5×10^-4^ mol/L) was pumped through the flow-through path. The fluorescence of reactant TBG containing solution was recorded at excitation and emission wavelength of 318 and 463 nm, respectively. An appropriate amount of H_2_O_2_ was then added to the same TBG solution giving a concentration of 2×10^-4^ mol/L H_2_O_2_. When the substrate solution containing TBG and H_2_O_2_ swept over the incubated biocomposite surface, the HRP of the BrAg-BrAb-HRP complexes bound on the support body surface catalyze the reaction to convert a part of TBG into a fluorescent dimmer product. The increase value in fluorescence intensity was recorded and taken as the evaluation of the amount of the BrAb analyte.

## 3. Results and Discussion

### 3.1. Properties of TBG

The stability of TBG, pHPPA, chavicol and Amplex red in air were examined. The results indicate that the fluorescence decrease rate of TBG was 3% after a period of 10 day-stored at room temperature, similar to that of pHPPA and chavicol. Amplex red exhibits insufficient stability. The sensitivity to temperature was tested with the substrates exposed to different temperatures from 37 to 80°C. The results showed that the fluorescence decrease rate of TBG was 8 % at 80°C, while the fluorescence decrease values for pHPPA, chavicol and Amplex red were 11.2%, 47% and 26%, respectively. This implies that TBG is sufficiently stable to the temperature tested.

The comparison studies on the response characteristics of TBG, pHPPA, chavicol and Amplex red reacting with H_2_O_2_ or H_2_O_2_/HRP were carried out, with the results shown in Table 1. One can notice that the response of TBG reacting with H_2_O_2_ is more lower than that of pHPPA, chavicol and Amplex red. The fluorescence response of TBG containing solution in the presence of H_2_O_2_/HRP is more significant than that of the other substrates examined. This implies that a more sensitive detection of HRP using TBG as a fluorogenic substrate can be realized due to the relatively low background signals deriving from the oxidation of H_2_O_2_.

### 3.2. Optimization of substrate concentration

The effect of TBG concentration on the fluorescence performance of HRP-enzymatic reaction solutions was investigated. To a B-R buffer solution (3 mL), 3×10^−4^mol/L H_2_O_2_-1.00×10^−6^ mol/L HRP-BrAb different concentrations of TBG were added. After a 2 min-reaction, the resulting solutions were subjected to fluorescence measurement. The results indicated that the fluorescence increases with the increase of the amount of TBG up to 5.00×10^−4^mol/L and then tended to saturate. A concentration of 5.0010^−4^mol/L TBG was optimal.

### 3.3. TBG-based Km determination for HRP

The *K_m_* for HRP using TBG as a substrate was evaluated. Only when the determination of enzymatic rate is at the initial rate, can the Michaelis equation be applied to detect a genuine *K_m_*. The *K_m_* for TBG-HRP-H_2_O_2_ system was measured by the initial velocity method. According to the countdown law, the fitting linear regression equation for TBG can be represented as follows:

$$1/v=5.5\times 10^{-13}\times 1/C_{\text{TBG}}+5\times 10^{-13}\;(r=0.998).$$

*V_max_* and *K_m_* can be given from the regression equation with a value of 2×10^2^.s ^−1^ and 1.1 ×10^−10^ mol/L, respectively. According to the same procedure, the *V_max_* for pHPPA, chavicol and Amplex red is 1.7×10^2^.s ^−1^, 12.1.s^−1^ and 1.3×10^2^.s^−1^, respectively. The results indicate that the reaction rate of TBG-HRP-H_2_O_2_ system is greater than that of the other substrates.

### 3.4. Characteristic performance of TBG fluorescence and quantitative basis

Stilbene glycoside, which acts in nature as an antioxidant in plants, was used fo the first time in the present BrAb assay as a fluorogenic substrate for HRP-catalyzed reaction. During the process HRP catalyses the dimerisation reaction of the 4-hydroxyphenyl-substituted compound TBG to form a fluorescent polyphenol compound. In the incubation procedure of the competitive binding immunoassay, the BrAb from the analytical samples and HRP-BrAb added would compete to bind on the supported BrAg biocomposite surface. The amount of HRP-BrAb binding on the supported biocomposite surface after incubation would be reversely proportional to the amount of BrAb in the analytical samples. The concentration of BrAb in samples (analyte) is consequently reversely proportional to the fluorescence increase of TBG solution.

**Figure 3.**
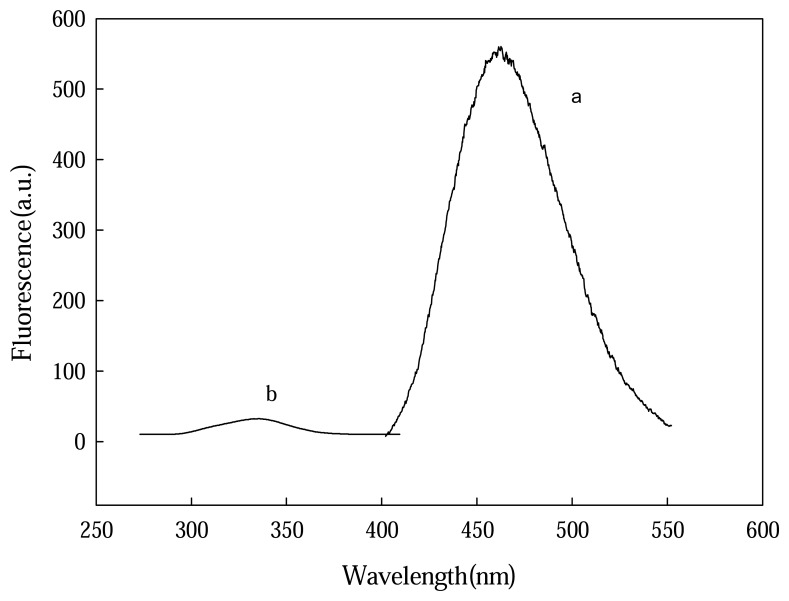
Fluorescence characteristics of TBG solution obtained by keeping the incubated immunocomposites (a) and the same immunocomposites without incubation (b) in a pH 5.8 B-R solution containing 3×10^-5^ mol/L TBG in the presence of 5×10^-4^ mol/L H_2_O_2_ for 1 min. Before this procedure, the renewed immunocomposite surface was subjected to a 30 min-incubation in a pH 5.8 B-R solution containing 5×10^-6^ g/L HRP-BrAb.

Fluorescence investigation of TBG was carried out in a HRP-BrAb-catalyzed reaction system. The excitation spectra of equilibrium solution showed that the λ_ex1_/λ_ex2_s of reactant TBG and its product were 250/305 nm and 277/318 nm, respectively (data not shown). The emission spectra of the equilibrium solution are shown in Figure 3. It was observed that a significant fluorescence peak occurred at 463 nm (λ_ex_ =318 nm) deriving from HRP-catalyzed products dimmers and its fluorescence intensity is dependent on HRP-BrAb concentration. This serves as the quantitative basis of the proposed sensing system for BrAb assay.

In contrast to the TBG substrate, no satisfactory result could be obtained with pHPPA and chavicol substrate due to small distance between the fluorescence peaks for reactant and product. The peak-peak distances obtained with pHPPA, chavicol and TBG with the same procedure were 102, 83 and 145 nm, respectively. The results showed that use of TBG for quantification is feasible with this approach. The response characteristics of TBG under the optimum experimental conditions seem to be also better than pHPPA, chavicol and Amplex red, so that TBG could be used as the right substrate in the present BrAb assay.

### 3.5. Optimization of experimental parameters

The selection of buffer solution such as B-R, HCl-Tris, Na_2_CO_3_-NaHCO_3_ and NaOH-H_3_BO_3_-KCl was carried out by measuring the fluorescence intensity of the HRP-enzymatic product of TBG. The results indicated that a sensitive response could be obtained with a B-R solution and it was employed as the reaction medium in most experiments.

Both the fluorescence of enzymatic product of TBG and the enzymatic reaction rate are affected by the pH value. The effect of pH on the TBG based enzymatic equilibrium solution was carried out. The results indicate that the fluorescence intensity of dimers keeps increasing with the increase of pH and reaches a peak at pH 5.8, and then begins to decrease, which may be associated with the fact that pH can influence the protonation of dimers.

The activity of enzyme label attached to the supported immunocomposites surface is affected by the pH of enzymatic reaction medium. A higher or lower pH of medium would inhibit the activity of enzyme. Additionally, the pH of the reaction medium has a strong effect on the oxidation of the catalytic product on the immunocomposites surface. The fluorescence response obtained increases with the increase in pH up to 5.8, then decreases with a maximum response at pH 6.5.

Generally, the optimal temperature of immunoreaction and enzymatic reaction would be 37 °C. At this temperature, however, a long incubation time would decrease the activity of the antigen, antibody and enzyme, leading to the deterioration of the response signals and a shorter lifetime of the immunocomposites. The results indicate that the signal increases with the increase of temperature up to 37 °C. An incubation and enzymatic reaction temperature of 35 °C was therefore adopted in most experiments.

### 3.6. Immunoassay for BrAb with proposed fluoroimmunosensing system

As a model system to demonstrate the utility of the novel HRP substrates in FELISA methods, BrAb, which is used as a indicator for *Brucellosis* diagnosis in the clinic and veterinary fields, was chosen as the detection target. The calibration curve for the determination of BrAb was established under the aforementioned optimal conditions. A good linear relationship was observed between the fluorescence intensity and BrAb concentration, and BrAb can be determined in the range of 3.5×10^−8^ g/L∼7.6 ×10^−6^ g/L.The linear regression equation was *y* = ‐3.2*x* + 645, with a correlation coefficient of 0.998 (*n* = 6), where *x* (BrAb) is in ×10^−8^ g/L. The detection limit (3σ) for BrAb was calculated from the standard deviation of the blank (*n* = 6) as 1.7×10^−9^g /L, which was on a par with that (7.6×10^−9^g/L) reported by the manufacturer based on FELISA. The results obtained were compared with those obtained by a conventional ELISA method in the clinical laboratory (Table 2). It indicates that the detectable concentration of BrAb using proposed immunosensing system meets the requirements of clinical analysis and can be used for the direct determination of BrAb concentration in rabbit serum.

In the same procedure, the fluoroimmunosensing determination of BrAb using pHPPA as the substrate for HRP was carried out. The pseudo-linear detection range for the BrAb assay extended between 7.2×10^−7^ g/L and 6.5 ×10^−5^ g/L with a detection limit of 1.2×10^−8^ g/L. This result demonstrates that the detection limit for TBG based fluoroimmunosensing system for BrAb determination can give a marked improvement, compared to those achieved with the substrate pHPPA using the same immunoreagent.

The reproducibility of the immunosensing system was tested with four regenerated biocomposites surface from the same batch. A series of eight measurements resulted in a relative standard deviation (RSD) of 3.8%. This means that the reproducibility results can be obtained using different surfaces of one signal immunosensing system.

### 3.7. HRP-catalyzed fluorogenic reaction mechanism

**Figure 4.**
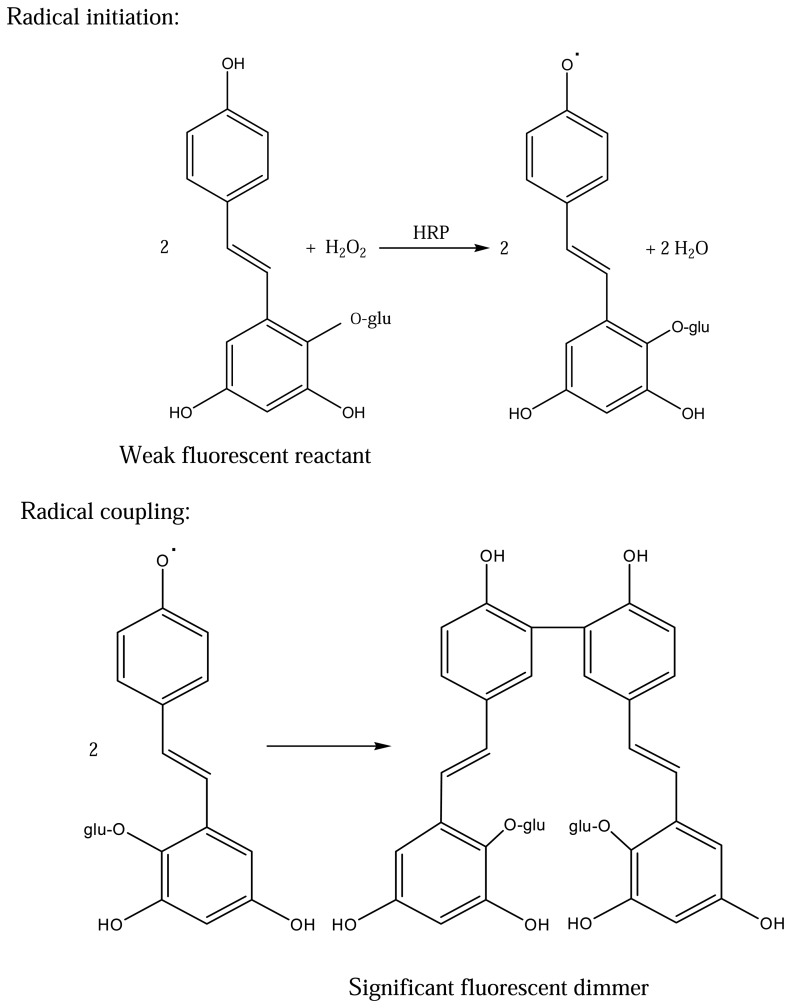
Mechanism skeleton of HRP-catalyzed reaction using TBG as fluorogenic substrate.

According to the literature [14, 15], it is well known that HRP catalyses the dimerisation of 4-hydroxyphenyl-substituted compounds, *e.g.* pHPPA, to form a fluorescent biphenol compound and enzymatic methods relying on this reaction are in widespread use. In our HRP-TBG system, HRP catalyses the polymerization reaction of TBG to form a fluorescent polyphenol compound which is similar to that of the phenyl substituted substrates reported.The proposed reaction mechanism for the HRP-TBG system is shown in Figure 4.

## 4. Conclusions

Stilbene glycoside is an alternative fluorogenic substrate for HRP detection for directly monitoring the concentration of BrAb in serum samples. The novel substrate TBG provides several advantages over commercially available substrates such as *p*-hydroxyphenylpropionic acid (pHPPA), chavicol and Amplex red. First, it exhibits satisfactory physical and chemical stability, low background fluorescence and a relatively sensitive response to HRP. Second, the HRP-linked immunosensing system based on the TBG gives a relatively sensitive and stable response with an HRP-BrAb tracer after a short incubation time. Finally, its good water-solubility make it easy to perform in the enzymatic reaction procedure and generate significantly more fluorescence response for HRP detection. This substrate TBG is a promising alternative to HRP-based FELISA methods for BrAb assay in the clinical laboratory.

## Figures and Tables

**Table 1.** Fluorescence response of TBG, pHPPA, chavicol and Amplex red solution with the addition of H_2_O_2_ or HRP-BrAb + H_2_O_2_

**Substrate** a	**Added H_2_O_2_^b^ (**Δ**F)**	**Added HRP+H_2_O_2_^b^ (**Δ**F)**
TBG	23	402
pHPPA	37	248
chavicol	62	179
Amplex red	116	161

a Concentration of substrate is 3 ×10^−5^ mol/L.

**Table 2.** BrAb determination with the proposed fluoroimmunosensing system in rabbit serum samples.

**Sample** a	**Proposed method (μg/L)**	**UV-ELISA (μg/L)**
1^#^	146±2.3^b^	144±2.4
2^#^	253±2.7	254±2.3
3^#^	457±2.1	460±2.6
4^#^	535±2.2	533±2.5

a The serum samples were diluted 200 times.

b Mean ± SD of four measurements.
